# Opportunities for near-surface small-angle neutron scattering to probe magnetic nanostructures within thin-film volumes

**DOI:** 10.1107/S1600576725005503

**Published:** 2025-07-22

**Authors:** Grace L. Causer

**Affiliations:** a School of Physics and Astronomy Monash University Clayton VIC 3800 Australia; b Australian Research Council Centre of Excellence in Future Low-Energy Electronics Technologies Monash University Clayton VIC 3800 Australia; Australian Centre for Neutron Scattering, ANSTO, Australia

**Keywords:** near-surface small-angle neutron scattering, grazing-incidence small-angle neutron scattering, magnetism, thin films

## Abstract

Near-surface small-angle neutron scattering (NS-SANS) is a highly versatile, yet under-utilized, technique in condensed matter research. It addresses the shortcomings of transmission SANS to enable the characterization of nano­structures within extremely small sample volumes in the thin-film limit. NS-SANS stands out in its capacity to resolve 1D, 2D or 3D structural, chemical and magnetic correlations beneath the surfaces of thin films with nanometre resolution. By varying the incident angle above the critical angle of reflection, NS-SANS delivers tuneable depth sensitivity across nano-confined volumes, effectively minimizing noise contributions from substrates while surpassing the surface-sensitive capabilities of grazing-incidence SANS. This perspective highlights the future potential of NS-SANS to study condensed matter thin films and heterostructures, with a special focus on nanoscale magnetic phenomena, such as topological skyrmion lattices, superconducting vortex lattices and chiral domain walls, which are of timely interest to the magnetism and quantum materials communities.

## Abstract 2

A perspective is given on the opportunities provided by near-surface small-angle neutron scattering for the characterization of nanoscale magnetic phenomena near surfaces and their extension into nano-confined volumes in the thin-film limit.

## 1. Introduction

Small-angle neutron scattering (SANS) is the method of choice for characterizing nanoscopic structural, chemical and magnetic correlations in bulk materials (Mühlbauer *et al.*, 2019 ▸). Resolving real-space length scales ranging from 1 to 300 nm requires high angular resolution, which is achieved on SANS instruments using long-distance collimation and pin­hole apertures. These design choices produce non-divergent and highly collimated beams with minimal background but limit the neutron flux at the sample position, leading to depleted signal intensities. As a result, conventional SANS measurements performed in transmission geometry rely on relatively large sample volumes, of the order of 1 mm^3^, to maintain adequate signal-to-background ratios.

By exploiting reflection geometries and enhanced scattering at glancing incidence (Jiang *et al.*, 2011 ▸), it becomes feasible to probe nanoscopic correlations in materials of significantly reduced sample volumes of the order of 10^−2^ mm^3^. Such volumes are typical of most solid-state thin films and heterostructures of nanometre thickness deposited on millimetre-squared substrates. As a result, reflection-mode SANS provides novel opportunities to characterize nanostructures within restricted sample volumes in the thin-film limit.

A key parameter in designing SANS experiments in reflection geometry is consideration of the incidence angle α_i_ (*i.e.* the angle subtended by the incident neutron beam from the film surface). The ratio between α_i_ and the critical angle of total external reflection of the material α_c_ determines the penetration depth of neutrons into the film and thus the sampling volume. When the incidence angle is chosen to be greater than or equal to the critical angle of the material, neutrons undergo small-angle scattering within the bulk volume of the film in a configuration known as near-surface SANS (NS-SANS) (Hamilton *et al.*, 1994 ▸; Hamilton *et al.*, 1996 ▸). Conversely, when α_i_ is chosen to be less than α_c_, neutrons undergo total external reflection at the sample surface to probe the topmost few atomic layers of the film only, in a configuration known as grazing-incidence SANS (GI-SANS) (Dosch, 1992 ▸).

By employing incident angles above the critical angle of reflection, NS-SANS extends beyond the mere surface sensitivity of GI-SANS to probe 1D, 2D and even 3D correlations beneath the surface of a material with tuneable depth sensitivity. Furthermore, by harnessing the magnetic sensitivity of neutrons, NS-SANS affords unique opportunities to study buried magnetic objects and related nanoscale magnetic phenomena within thin films and micro-structured bulk materials. To date, NS-SANS has been employed in the field of soft matter for the study of polymer micelles (Hamilton *et al.*, 2005 ▸; Hamilton *et al.*, 1996 ▸; Kyrey *et al.*, 2021 ▸; Ruderer *et al.*, 2012 ▸; Wolff *et al.*, 2007 ▸) but is yet to be comprehensively reported on for the study of magnetic nanostructures in low-dimensional solid-state materials.

This perspective discusses the opportunities for NS-SANS investigations of nano-confined systems in condensed matter research. Special attention is paid to sub-surface magnetic phenomena representing fundamental open issues in con­tem­porary physics, such as flux-line lattices in type II superconductors, non-collinear spin textures in non-centrosymmetric magnets and chiral domain walls in heavy-metal ferromagnetic multilayers. Details of the NS-SANS geometry are presented, and the importance of the choice of incidence angle is discussed. The advantages of NS-SANS over popular experimental probes, such as Lorentz transmission electron microscopy and polarized neutron reflectometry, are also outlined.

## 2. Description of the NS-SANS geometry

**Figure 1:**
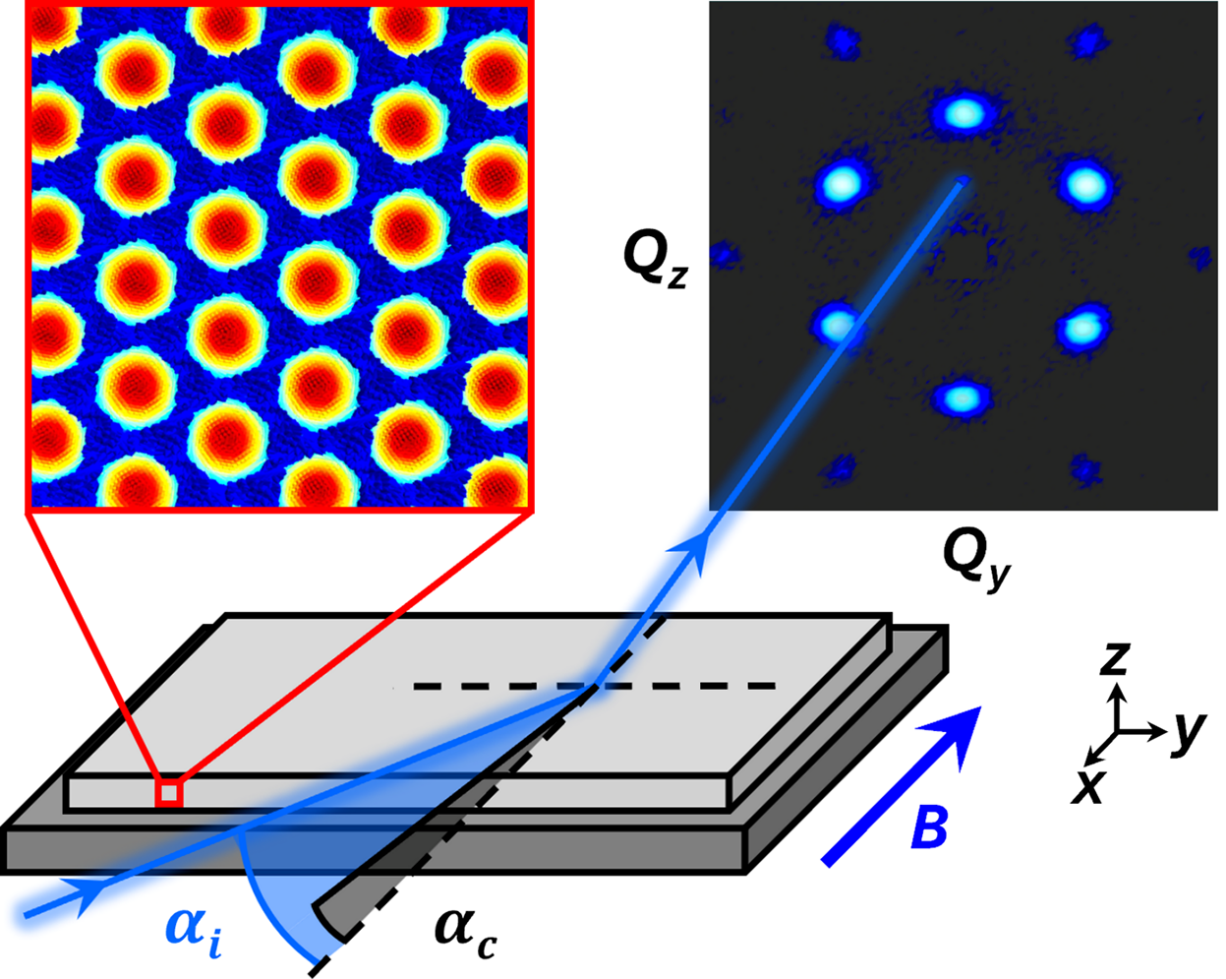
Schematic of the NS-SANS geometry for a vertical reflection plane. The coordinate system is chosen such that the sample surface lies in the *xy* plane and the scattering plane of the detector lies in the *yz* plane. A beam of monochromatic and highly collimated neutrons propagates along the **x** direction and impinges onto the sample surface at a shallow incidence angle α_i_ greater than or equal to the critical angle α_c_ of the sample. In this geometry, the sample is fully illuminated and the bulk ordering of the sample is probed. The resulting detector image will be sensitive to lateral and vertical correlations in *Q*_*y*_ and *Q*_*z*_, respectively.

Fig. 1 ▸ depicts the NS-SANS geometry for a vertical reflection plane. A highly collimated beam of monochromatic neutrons propagates along the **x** direction and impinges onto the sample surface at a shallow incidence angle α_i_ greater than or equal to the critical angle α_c_ of the sample. The sample’s α_c_(= 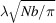) is determined by its scattering length density *Nb*, resulting in typical α_c_ values of less than 1° due to the refractive index of most materials being close to 1. A neutron detector located in the *yz* scattering plane is sensitive to lateral and vertical structures in the *yz* sample plane. Additional details of the NS-SANS setup are described elsewhere (Causer *et al.*, 2023*a* ▸).

For samples exhibiting long-range magnetic order that orients along magnetic field lines, such as the skyrmion lattice depicted in Fig. 1 ▸, an in-plane magnetic field applied parallel to the neutron beam along the **x** direction will arrange the hexagonal lattice within the *yz* sample plane, and the resulting detector image will be sensitive to lateral and vertical correlations in *Q*_*y*_ and *Q*_*z*_, respectively. Conversely, an in-plane magnetic field applied perpendicular to the neutron beam along the **y** direction will arrange the hexagonal lattice within the *xz* sample plane, allowing lateral correlations in *Q*_*x*_ to be probed as well, which are otherwise nominally ordered along the neutron beam in parallel fields. The locations of the magnetic Bragg peaks on the detector will be independent of the chosen α_i_ but may exhibit field and temperature dependencies, and their sharpness will be influenced by the range of the magnetic order (*i.e.* short range or long range). A specular peak will arise on the detector at α_i_ = α_f_ which will depend on the neutron wavelength but will be independent of temperature and field. The sharpness of the specular peak will be influenced by the roughness of the sample surface and its intensity will be governed by the neutron absorption cross section. To account for dynamic effects at glancing incidence, quantitative analysis of NS-SANS data is performed within the framework of the distorted-wave Born approximation, which is integrated into open-source software analysis programs such as *BornAgain* (Pospelov *et al.*, 2020 ▸).

**Figure 2:**
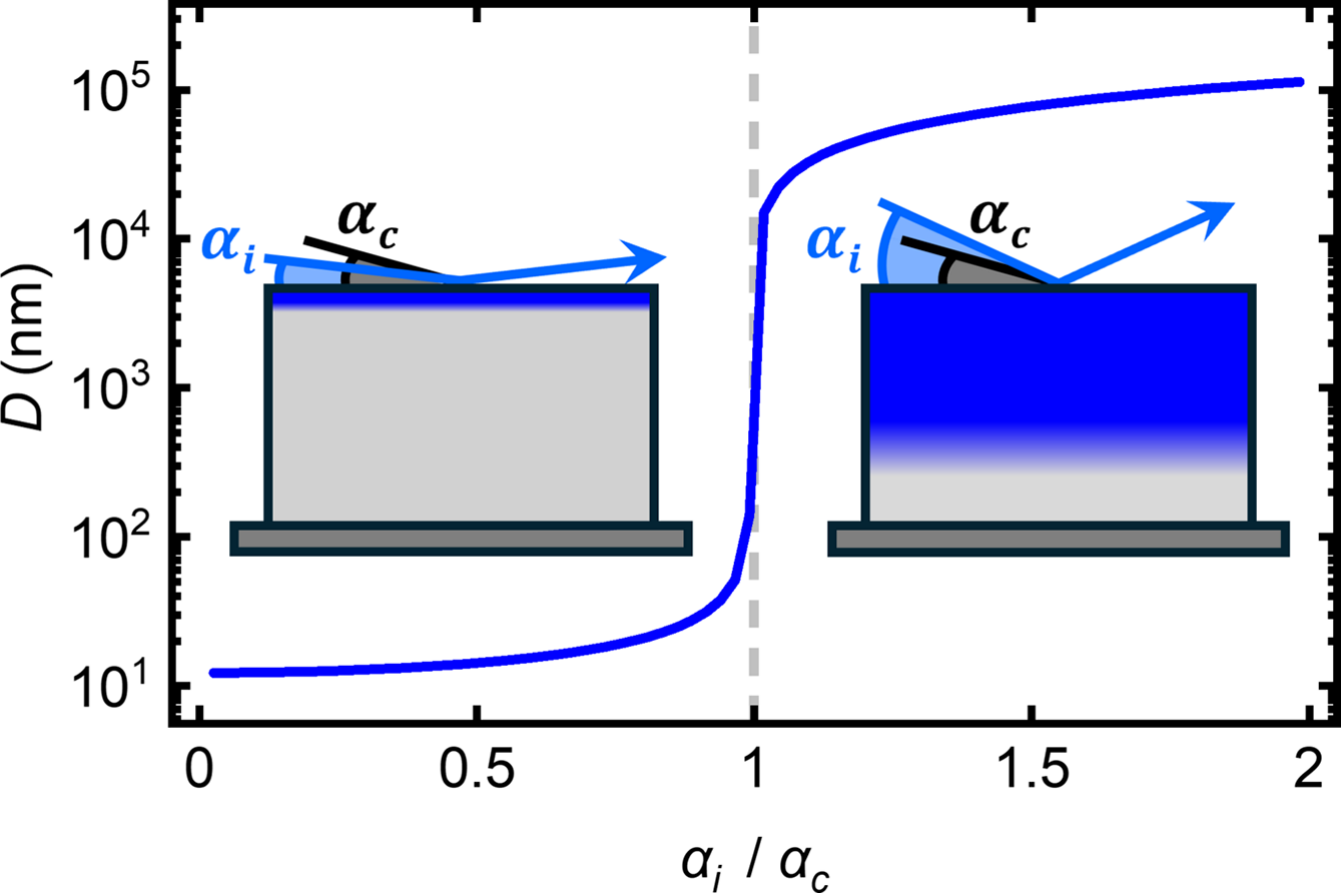
Penetration depth *D* of neutrons as a function of α_i_/α_c_. The exemplar curve has been calculated for the cubic chiral magnet Fe_0.75_Co_0.25_Si at a neutron wavelength of 5 Å, resulting in a critical angle of α_c_ = 0.37°. Depending on the material species, the absolute values of *D* and α_i_/α_c_ will vary and can be determined from equations (1) and (2). For α_i_/α_c_ = 1, neutrons begin to undergo small-angle scattering within the bulk volume of the film in the NS-SANS regime. For increasingly larger incident angles, such that α_i_/α_c_ > 1, the penetration depth of neutrons into the sample is greatly enhanced and NS-SANS measurements can be optimized towards bulk sensitivity in the range of 10^4^ nm below the sample surface.

In the reflection geometry, the penetration depth *D* of neutrons into the sample is well documented (Dosch, 1992 ▸; Müller-Buschbaum, 2013 ▸) and is given by 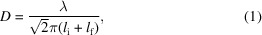
where 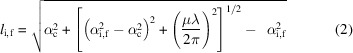
and μ is the attenuation coefficient. Plotting *D* versus α_i_/α_c_ yields a characteristic S-shaped profile as depicted in Fig. 2 ▸. When α_i_ is equal to α_c_, such that α_i_/α_c_ = 1, the sample is fully illuminated and neutrons begin to undergo small-angle scattering within the bulk volume of the film in the NS-SANS regime. For increasingly larger incident angles, such that α_i_/α_c_ > 1, the penetration depth of neutrons into the sample is greatly enhanced and the NS-SANS measurements can be optimized towards bulk sensitivity in the range of up to 10^4^ nm below the sample surface.

## 3. Opportunities for NS-SANS in condensed matter research

The standardization of the NS-SANS technique and its adoption into the everyday vernacular of condensed matter researchers have the potential to increase the number of experimental investigations carried out on thin-film and heterostructure samples. Furthermore, owing to the neutron’s spin degree of freedom, NS-SANS is likely to lead to several advances in the scientific understanding of magnetic phenomena near surfaces and their extension into nano-confined volumes, resulting in breakthroughs in many areas of magnetism research. The opportunities of NS-SANS in condensed matter research are discussed below.

### 3.1. NS-SANS probes nanoscale periodicities within nano-confined volumes

It is well understood that conventional SANS measurements are impractical for investigating thin films and heterostructures, as their scattering volumes are too small to be measured in transmission (Meynell *et al.*, 2017 ▸). Early attempts to address this problem focused on stacking several identically prepared films in transmission to enhance the signal-to-background ratio (Farmer *et al.*, 2019 ▸; Desautels *et al.*, 2019 ▸). However, this approach requires the preparation of many identical and co-aligned films, where small misalignments in the crystallographic axes between adjacent films in the stack may result in an unwanted smearing of diffraction peaks, leading to ambiguity in the data.

In contrast, the glancing-incidence geometry of NS-SANS creates an extended path length for neutrons within the film, increasing the effective scattering volume compared with the transmission geometry. Hence, NS-SANS makes it possible to probe mesoscopic correlations by small-angle scattering within the whole volume of a single thin-film specimen (and not a stack of films), with good data statistics achieved within a feasible counting time of a few hours.

**Figure 3:**
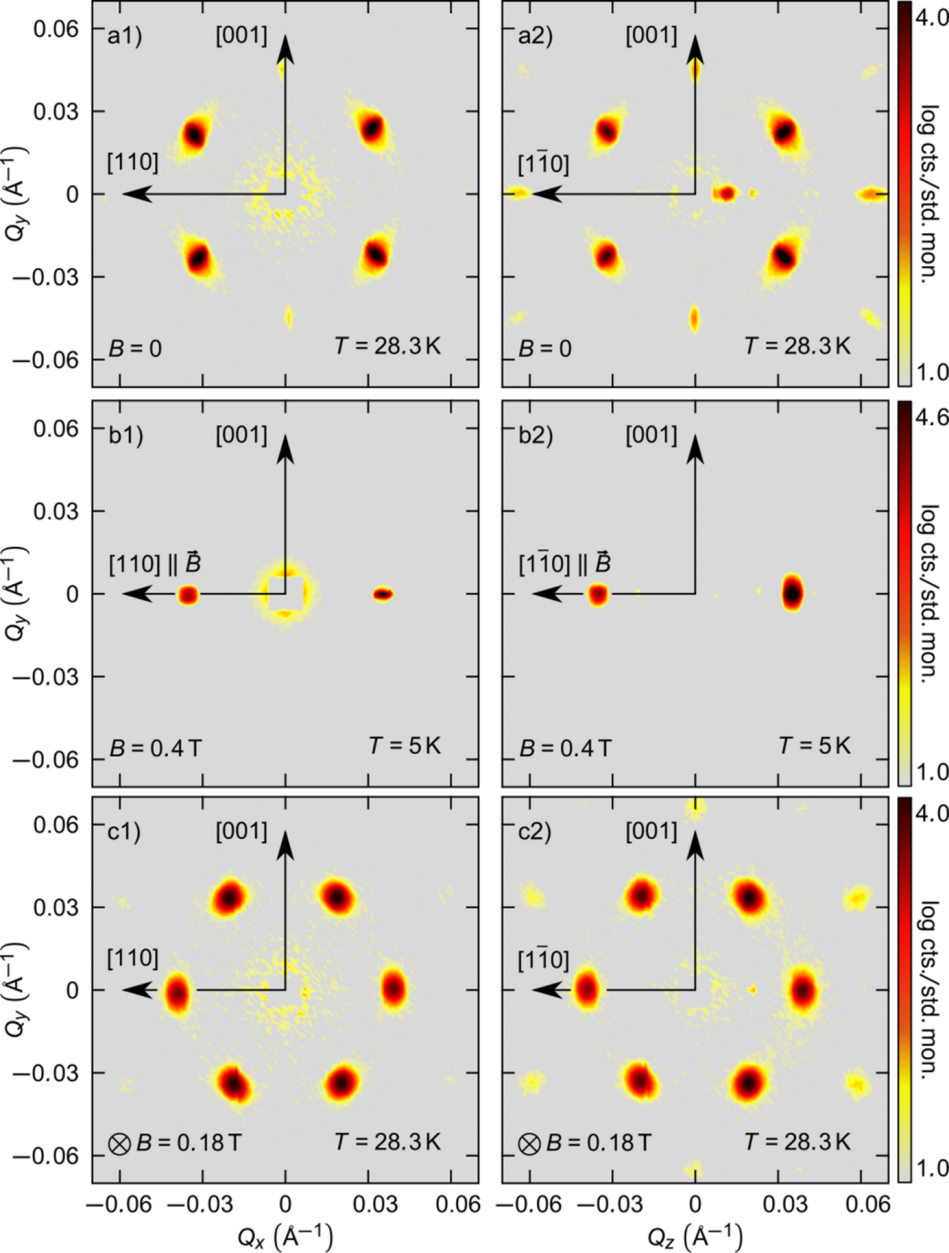
Comparison of scattering patterns obtained in the (*a*) helical, (*b*) conical and (*c*) skyrmion lattice phases of MnSi in the transmission SANS geometry (column 1) and in the NS-SANS geometry (column 2). Adapted with permission from Causer *et al.* (2023*a* ▸).

By illuminating samples above their critical angle of reflection, NS-SANS is sensitive to periodic nanoscale structures within the sub-surface region of restricted sample volumes. The data presented in Fig. 3 ▸ [adapted from Causer *et al.* (2023*a* ▸)] illustrate experimental scattering patterns obtained for a bulk sample measured in transmission geometry and a polished surface of the same bulk crystal measured in the NS-SANS geometry. The scattering patterns obtained in the NS-SANS geometry match those obtained in transmission, albeit with the addition of specular scattering. This consistency validates the effectiveness of NS-SANS, demonstrating that, despite its modified scattering geometry compared with transmission SANS, it probes the bulk volume of a sample to provide a comprehensive depiction of the nanoscale periodicities present within nano-confined volumes.

It is of scientific interest to investigate the properties of materials prepared in reduced dimensions, such as thin films and micro-structured bulk materials, as growing evidence suggests that their magnetic properties differ substantially from the properties of genuine bulk materials. Typical open questions may be nicely illustrated in the class of cubic chiral magnets, where the magnetic phase diagrams differ distinctly between bulk single crystals and epitaxial films (Wolf *et al.*, 2022 ▸; Wiedemann *et al.*, 2017 ▸; Huang & Chien, 2012 ▸; Karhu *et al.*, 2011 ▸; Park *et al.*, 2014 ▸; Wilson *et al.*, 2013 ▸; Yu *et al.*, 2011 ▸; Yokouchi *et al.*, 2015 ▸). Hence, there is a unique opportunity to exploit the broad reach of SANS techniques and bridge the gap between bulk studies conducted in transmission and thin-film studies conducted in reflection.

### 3.2. NS-SANS is non-destructive and provides average statistical information

Real-space imaging techniques, such as Lorentz transmission electron microscopy and electron holography (Wolf *et al.*, 2022 ▸; Yu *et al.*, 2018 ▸), are some of the most common methods for studying magnetic correlations at and beneath the surfaces of materials of reduced sample dimension. However, information is provided on local scales only (typically tens to hundreds of nanometres) and their applicability to extended films and heterostructures supported by substrates is limited (Li *et al.*, 2013 ▸; Heinze *et al.*, 2011 ▸; Milde *et al.*, 2013 ▸). This is because samples are required to be electron transparent, such that either substrates are mechanically milled away from films of interest or films of interest are chemically exfoliated from their substrates. Both approaches can deform thin-film lattices, introducing new stresses and strains and altering their electronic and magnetic properties (Venuti *et al.*, 2024 ▸).

In view of these limitations, a significant advantage of NS-SANS is that it is a non-destructive probe, in the sense that it does not cause radiation damage to the sample and allows one to study a film in its ‘pristine’ state in the presence of its substrate. As a result, no damage is caused to thin-film lattices studied by NS-SANS, ensuring that native properties are preserved and investigated. Furthermore, the large footprint of the neutron beam on the sample surface at glancing incidence in the NS-SANS geometry ensures that reciprocal-space information is averaged over macroscopic volumes, allowing comprehensive analysis of the global structure, rather than the local structure, of the material under investigation (Müller-Buschbaum, 2013 ▸).

### 3.3. NS-SANS resolves 1D, 2D or 3D structures with nanometre resolution

As the method employs a highly collimated beam, NS-SANS can be used to probe the internal magnetic order of a film with nanometre resolution along each sample axis (*x*, *y* and *z*). In a similar vein, NS-SANS can be used to ascertain the dimensionality (1D, 2D or 3D) of unknown magnetic structures within thin films and near the surfaces of bulk materials, provided the structure has magnetization components normal to the scattering vector. Particularly elegant examples pertain to structures which orient along magnetic field lines, such as superconducting vortices and skyrmion lattices (Brems *et al.*, 2022 ▸; Bishop *et al.*, 1992 ▸; Mühlbauer *et al.*, 2009 ▸; Adams *et al.*, 2018 ▸). Here, a standard horizontal magnet capable of applying magnetic fields parallel and perpendicular to the neutron beam will orient these lattices in front-on orientations (as in Fig. 1 ▸) and side-on orientations, respectively, allowing reciprocal-space information in *Q*_*x*_, *Q*_*y*_ and *Q*_*z*_ to be obtained. Similarly, NS-SANS is equally adept at resolving structural or chemical periodicities (*i.e.* non-magnetic-field-dependent periodicities) in *Q*_*x*_, *Q*_*y*_ and *Q*_*z*_ by rotating the in-plane sample axes 90° relative to the neutron beam between two consecutive measurements (note that *Q*_*z*_ information is contained in each measurement).

Importantly, the nanometre-scale resolution achievable with NS-SANS surpasses the resolving power attainable by polarized neutron reflectometry (PNR). PNR is considered the most well established neutron scattering technique for thin-film analysis (Causer *et al.*, 2023*b* ▸), but it is limited to nanometre resolution in *Q*_*z*_, micrometre resolution in *Q*_*x*_ (parallel to the neutron direction) and no resolution in *Q*_*y*_ (perpendicular to the neutron direction) due to relaxed beam collimation along *Q*_*y*_ (Saerbeck, 2014 ▸). Thus, NS-SANS is a far superior technique enabling comprehensive 3D mapping of magnetic, structural or chemical periodicities in nano-confined materials, and it is particularly indispensable when fine nanoscale analysis across all spatial dimensions is required.

### 3.4. NS-SANS surpasses the surface-only sensitivity of GI-SANS

It is widely acknowledged that the emergent magnetic phenomena exhibited by most magnetic materials occur beneath their surfaces, extending across their entire thickness. Examples include skyrmion tubes in cubic chiral magnets (Mühlbauer *et al.*, 2009 ▸), quintuple-layer antiferromagnetic order in magnetic topological insulators (Li *et al.*, 2023 ▸), magnetic fan-like structures in oxide heterostructures (Guasco *et al.*, 2022 ▸), chiral soliton lattices in monoaxial chiral magnets (Kishine & Ovchinnikov, 2015 ▸), magnetic vortices in altermagnets (Amin *et al.*, 2024 ▸), flux-line lattices in type II superconductors (Brems *et al.*, 2024 ▸), Bloch skyrmions in nano-pillar arrays (Gilbert *et al.*, 2015 ▸) and vertical chiral domain walls in heavy-metal ferromagnetic heterostructures (Stellhorn *et al.*, 2019 ▸).

The surfaces of bulk magnets appear to reflect changes in the energetics of samples with reduced dimension. Chiral bobbers and non-trivial 3D hopfions have been reported at the surfaces of thinned bulk compounds (Redies *et al.*, 2019 ▸; Zheng *et al.*, 2023 ▸). Ferromagnetism has been observed near the surfaces of non-magnetic bulk crystals (Ohtsuka *et al.*, 2021 ▸; Jaccarino *et al.*, 1967 ▸), and the surfaces of B20 magnets support an unexplained strong evolution from Néel to Bloch twisting (Zhang *et al.*, 2018 ▸). These results in combination demonstrate the need for sub-surface probes capable of resolving nanoscale magnetic structures with nanometre to micrometre spatial extent beneath the surfaces of nano-confined volumes.

As NS-SANS illuminates samples above their critical angle of total external reflection, sub-surface correlations within the bulk volume of the sample are probed. This characteristic makes NS-SANS a more comprehensive and universal technique for nanostructure determination compared with GI-SANS. In the latter configuration, samples are illuminated below their critical angle of total external reflection and only an evanescent wave extends into the sample volume, which is exponentially damped within a few nanometres of the surface. As a result, GI-SANS is suited to probing surface roughness or the size and shape of nanostructures (such as nano-dot or nano-stripe arrays) positioned at or on top of a surface (Nouhi *et al.*, 2017 ▸).

Nevertheless, it can be challenging to achieve pure GI-SANS conditions in an experiment, as the GI-SANS condition requires incident angles less than the critical angle of reflection. Table 1 ▸ lists the critical angles, scattering length densities (SLDs) and correlation lengths (*L*) of a select number of monoaxial chiral magnets, cubic chiral magnets and type II superconducting bulk materials of interest. It is observed that α_c_ for all materials listed in the table is less than 0.5° at a neutron wavelength of 5 Å. The problem is exacerbated in low-SLD materials such as MnSi (with SLD = 0.18 × 10^−6^ Å^−2^) which has a critical angle of 0.069° at 5 Å; this can be difficult to achieve experimentally.

Distinguishing between GI-SANS and NS-SANS regimes becomes problematic for magnetic samples measured near α_i_/α_c_ = 1. The prevalence of spin-dependent critical angles at magnetic surfaces and spin-flip scattering at magnetic interfaces suggests the potential to superimpose NS-SANS and GI-SANS conditions in a single measurement. For instance, in the case of an unpolarized beam incident on a magnetic sample near α_c_, the spin-up component may be incident below its α_c_ in the GI-SANS regime, while the spin-down component might be incident above its α_c_ in the NS-SANS regime. Scenarios such as this suggest that regime classification should consider the characteristics of both incoming and outgoing spin channels, and this is a matter for future investigation.

### 3.5. NS-SANS provides tuneable sampling depth

By selectively varying the incident angle above the critical angle of reflection, the NS-SANS regime provides tuneable depth sensitivity across nano-confined volumes. Consequently, this provides NS-SANS with the functionality to avoid scattering from substrates and support structures, which otherwise form a source of background in transmission SANS measurements.

For a homogeneous sample, the penetration depth of neutrons at a given incidence angle will depend on the attenuation and α_c_ of the material, as outlined in equations (1) and (2). The neutron wavelength can be used as an additional control parameter of the penetration depth, as the α_c_ of a given material will vary linearly with λ. For an inhomogeneous sample, such as a heterostructure, the situation becomes more complex as the beam is modulated by multiple reflections and refractions at the thin-film interfaces. Nonetheless, typical penetration depths ranging from nanometres to micrometres are achieved in the NS-SANS regime, as shown in Fig. 2 ▸. Accordingly, NS-SANS can be used to investigate films of differing thicknesses, as well as to perform volume-dependent characterizations of the average sub-surface structure in single specimens.

### 3.6. Polarized NS-SANS enables characterization of chiral nanostructures

Polarized NS-SANS experiments provide opportunities to improve our understanding of thin-film chiral spin systems. Opportunities exist to study the chirality of non-collinear spin textures characterized by continuous left-handed or right-handed rotations of the sample’s magnetic moments in non-centrosymmetric magnets. Examples include helical and conical spin textures in cubic chiral magnets such as MnSi, FeGe, MnGe, Fe_1−*x*_Co_*x*_Si and Cu_2_OSeO_3_ (Bauer & Pfleiderer, 2016 ▸), as well as chiral soliton lattices in monoaxial chiral magnets such as CrNb_3_S_6_ and YbNi_3_Al_9_ (Kishine & Ovchinnikov, 2015 ▸).

Polarized NS-SANS could be more broadly employed to characterize the chirality of topological features including Néel and Bloch domain walls (Gilbert *et al.*, 2015 ▸; Liyanage *et al.*, 2023 ▸), as recently demonstrated in ferromagnetic heavy-metal heterostructures (Stellhorn *et al.*, 2019 ▸). Moreover, polarized NS-SANS facilitates the separation of nuclear and magnetic scattering contributions, enabling a clearer analysis of the depth and lateral distribution of spin textures and their evolution under external stimuli, such as magnetic fields and temperature variations. These capabilities would be invaluable for advancing research in spintronics, quantum computing and materials exhibiting exotic magnetic phases.

## 4. Conclusion

This article presents a perspective on the benefits of NS-SANS geometries for the characterization of nanoscale magnetic structures and related phenomena in condensed matter systems in the thin-film limit. In summary, NS-SANS is a comprehensive scattering technique providing access to 1D, 2D and 3D structures located beneath the surfaces of mater­ials. It offers tuneable depth sensitivity and provides information which is averaged over macroscopic volumes, enabling global structure determination. Given that transmission SANS is subject to prohibitively high background and the conditions for GI-SANS can be difficult to satisfy, NS-SANS is likely to lead to several advances in the scientific understanding of magnetic phenomena near surfaces and their extension into nano-confined volumes, resulting in breakthroughs in many areas of magnetism research.

## Figures and Tables

**Table 1:** Neutron SLDs and the corresponding critical angles α_c_ for selected bulk monoaxial chiral magnets, cubic chiral magnets and type II superconductors Values of the critical angle (α_c_ = 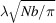) are calculated for a neutron wavelength of 5 Å. The correlation lengths *L* correspond to monoaxial chiral magnets in the zero-field helical phase, cubic chiral magnets in the skymion phase and type II superconductors in the Shubnikov phase. The magnetic field and temperature settings of each material’s *L* are provided.

	Neutron SLD (× 10^−6^ Å^−2^)	α_c_ at 5 Å (°)	*L* (nm)	Reference
*Monoaxial chiral magnets*				
CrNb_3_S_6_	2.44	0.25	48 (100 K, 0 T)	Song *et al.* (2020 ▸)
CsCuCl_3_	3.10	0.28	22 (10 K, 0 T)	Adachi *et al.* (1980 ▸)
YbNi_3_Al_9_	3.64	0.42	34 (3 K, 0 T)	Ohara *et al.* (2014 ▸)

*Cubic chiral magnets*				
MnSi	0.18	0.069	18 (28.5 K, 0.15 T)	Mühlbauer *et al.* (2009 ▸)
FeGe	6.73	0.42	70 (278 K, 0.02 T)	Siegfried *et al.* (2017 ▸)
Fe_0.75_Co_0.25_Si	5.30	0.37	30 (34 K, 0.05 T)	Bauer *et al.* (2016 ▸)
MnGe	1.61	0.21	6 (170 K, 0.2 T)	Kanazawa *et al.* (2011 ▸)
Cu_2_OSeO_3_	5.25	0.37	62 (57 K, 0.04 T)	Adams *et al.* (2012 ▸)

*Type II superconductors*				
Nb	3.92	0.32	125 (4 K, 0.15 T)	Riemann *et al.* (2011 ▸)
PbIn	2.37	0.25	600 (1.2 K, 0.007 T)	Brandt (1995 ▸)
YBa_2_Cu_3_O_7−δ_	4.65	0.35	100 (20 K, 0.2 T)	Forgan *et al.* (1990 ▸)
Bi_2.15_Sr_1.95_CaCu_2_O_8+*x*_	4.30	0.34	300 (1.5 K, 0.02 T)	Cubitt *et al.* (1993 ▸)
